# Multi-scale imaging of anticancer platinum(iv) compounds in murine tumor and kidney

**DOI:** 10.1039/c5sc04383b

**Published:** 2016-02-03

**Authors:** A. A. Legin, S. Theiner, A. Schintlmeister, S. Reipert, P. Heffeter, M. A. Jakupec, J. Mayr, H. P. Varbanov, C. R. Kowol, M. S. Galanski, W. Berger, M. Wagner, B. K. Keppler

**Affiliations:** Institute of Inorganic Chemistry, Research Platform “Translational Cancer Therapy Research,” and Research Network “Chemistry meets Microbiology”, University of Vienna Währinger Straße 42 A-1090 Vienna Austria bernhard.keppler@univie.ac.at +43-1-4277-52600; Department of Microbiology and Ecosystem Science, Research Network “Chemistry meets Microbiology”, and Large-Instrument Facility for Advanced Isotope Research, University of Vienna A-1090 Vienna Austria; Core Facility of Cell Imaging and Ultrastructure Research, University of Vienna A-1090 Vienna Austria; Institute of Cancer Research, Comprehensive Cancer Center and Research Platform “Translational Cancer Therapy Research”, Medical University of Vienna A-1090 Vienna Austria

## Abstract

Nano-scale secondary ion mass spectrometry (NanoSIMS) enables trace element and isotope analyses with high spatial resolution. This unique capability has recently been exploited in several studies analyzing the subcellular distribution of Au and Pt anticancer compounds. However, these studies were restricted to cell culture systems. To explore the applicability to the *in vivo* setting, we developed a combined imaging approach consisting of laser ablation inductively coupled plasma mass spectrometry (LA-ICP-MS), NanoSIMS and transmission electron microscopy (TEM) suitable for multi-scale detection of the platinum distribution in tissues. Applying this approach to kidney and tumor samples upon administration of selected platinum(iv) anticancer prodrugs revealed uneven platinum distributions on both the organ and subcellular scales. Spatial platinum accumulation patterns were quantitatively assessed by LA-ICP-MS in histologically heterogeneous organs (*e.g.*, higher platinum accumulation in kidney cortex than in medulla) and used to select regions of interest for subcellular-scale imaging with NanoSIMS. These analyses revealed cytoplasmic sulfur-rich organelles accumulating platinum in both kidney and malignant cells. Those in the tumor were subsequently identified as organelles of lysosomal origin, demonstrating the potential of the combinatorial approach for investigating therapeutically relevant drug concentrations on a submicrometer scale.

## Abstract 2

A NanoSIMS, LA-ICP-MS and TEM combinatorial approach was applied to analyze the distribution of platinum(iv) prodrugs in murine tissue samples.

## Graphical abstract

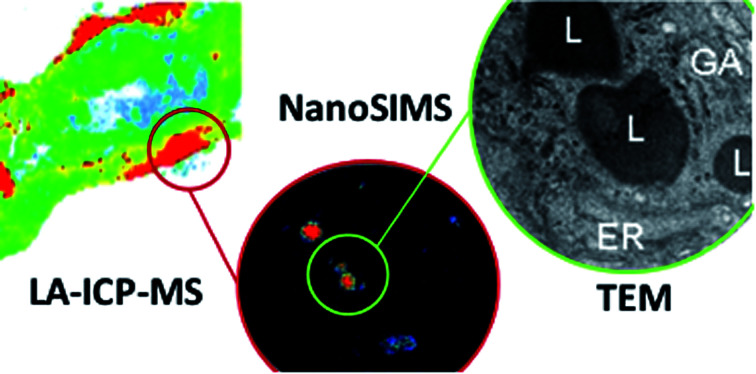

## Introduction

Platinum-based drugs play a pivotal role in cancer chemotherapy and are clinically used for treatment of a variety of solid tumors, including testicular, colorectal, bladder, ovarian, as well as head and neck cancer.^1^ Cisplatin, carboplatin, and oxaliplatin are the most frequently applied platinum drugs, even though the mechanisms underlying their efficacy, side effects and partially different activity profiles are not sufficiently well understood. Due to the lack of proper technologies, the investigation of the intracellular fate of platinum compounds in healthy and malignant tissues *in vivo* had been a poorly studied aspect in metal-based anticancer drug research. Knowledge on the distribution of these drugs in tumors and other tissues at multiple scales of resolution could substantially contribute to our understanding of their biological activity, cancer selectivity and toxicity. The subcellular distribution of cisplatin was previously studied in adherent cell cultures.^2^ However, imaging the distribution of this compound in animal tissues is a highly challenging task due to the low accumulation of cisplatin in murine samples at the maximum tolerated dose (Table 1). For developing an approach that enables platinum drug imaging in tissues, we therefore selected compounds with high tissue accumulation facilitating analysis under *in vivo* settings.

**Table 1:** Average platinum concentrations in tumor, kidney and liver samples of treated mice determined by ICP-MS (values are presented as mean ± standard deviation; i.p. - intraperitoneal, i.v. - intravenous)

Compound	Route of admin.	Pt concentration [μg g^−1^]		
		Tumor	Kidney	Liver
1^a^	i.p.	1.29 ± 0.24	8.15 ± 0.38	12.35 ± 1.47
2	i.v.	4.05 ± 0.32	4.87 ± 0.45	3.20 ± 0.13
Cisplatin	i.v.	0.25 ± 0.05	1.67 ± 0.36	1.26 ± 0.15

a Data taken from ref. 7.

**Fig. 1:**
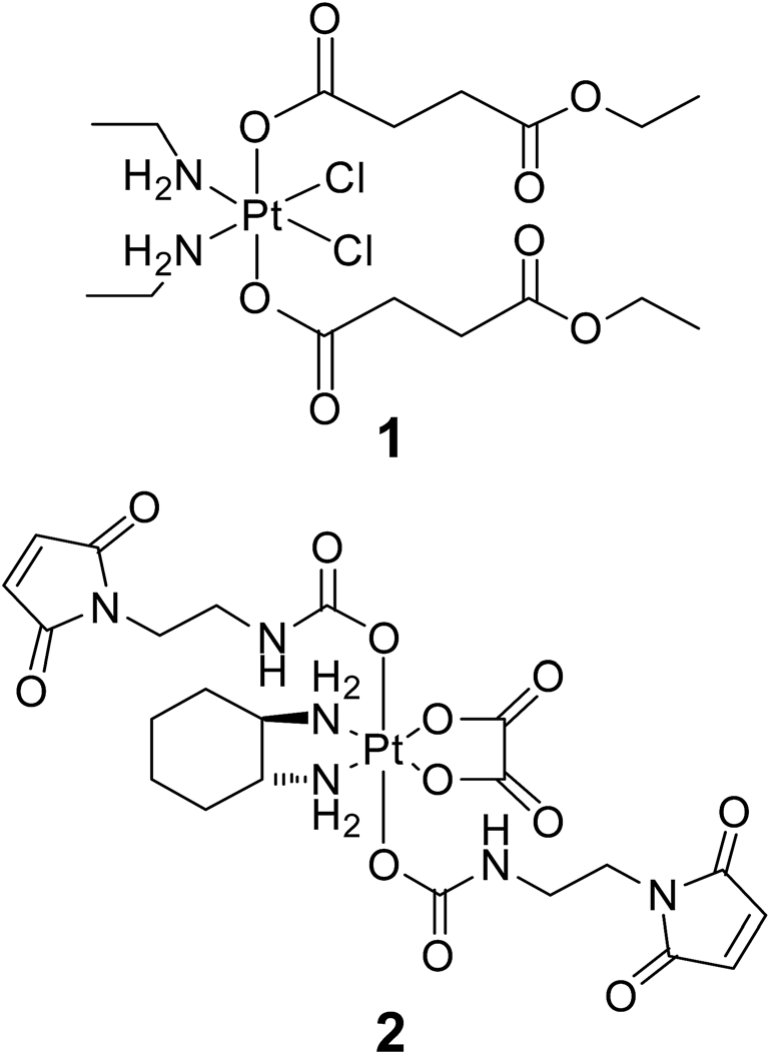
Structural formulas of investigated platinum(iv)-based anticancer compounds.

Platinum(iv) compounds are designed as prodrugs with potentially improved pharmacological properties in comparison to platinum(ii) anticancer agents, based on site-specific activation in the tumor tissue.^3,4^ Octahedral platinum(iv) complexes are kinetically inert to ligand-exchange reactions and their active square-planar platinum(ii) metabolites are released only after reduction (*e.g.*, by ascorbic acid, glutathione, or high-molecular-weight reducing agents).^5,6^ In addition to site-specific activation, variation of the axial ligands enables fine-tuning of pharmacologically relevant physicochemical properties (*e.g.*, lipophilicity, redox behavior). From many substances tested, a novel bis(carboxylato)dichloridoplatinum(IV) and a tetracarboxylatoplatinum(IV) complex (further referred to as compounds 1 and 2, Fig. 1) showed higher accumulation in kidney and tumor (Table 1), respectively, than any other compound and, therefore, were chosen for analysis in the respective tissue. Moreover, higher Pt accumulation than in the case of the clinically established drugs cisplatin or oxaliplatin *in vivo*^7–9^ render them promising candidates for drug distribution studies by chemical imaging.

Compound 1 belongs to a set of platinum(iv) complexes that was previously extensively optimized with regard to their solubility, lipophilicity and redox behavior.^10–12^ Compound 1 demonstrated high cytotoxicity *in vitro* (comparable to or more potent than cisplatin), together with adequate water solubility and lipophilicity in the optimal range for oral application (log *P*_o/w_ 0.5–3.5).^13^ Uptake experiments in cell cultures and therapeutic tests in mice revealed a high platinum accumulation in bulk analyses of cells and organs, respectively.^7^

Compound 2 belongs to a class of novel oxaliplatin-based maleimide-functionalized Pt(iv) complexes that were synthesized as precursors for binding to thiol-containing tumor-targeting molecules such as human serum albumin (HSA).^8^ The targeting strategy is based on the accumulation of albumin, the most abundant serum protein, in tumor tissues due to the enhanced permeability and retention (EPR) effect.^14^ The principle of tumor targeting by drug–HSA conjugates was already proven in several clinical studies for organic chemotherapeutics^15,16^ and became clinically established with the approval of nanoparticle albumin-bound paclitaxel in 2005.

Nano-scale secondary ion mass spectrometry (NanoSIMS) is increasingly being used for multi-elemental, isotope-selective imaging of isotopically labeled compounds^17–21^ as well as rare elements (*e.g.*, noble metals)^2,22,23^ in biological samples with complex chemical composition. These studies benefit particularly from the combination of excellent spatial resolution (down to 50 nm) with high sensitivity and high mass resolution offered by NanoSIMS. In the field of chemical pharmacology, researchers have now begun to explore NanoSIMS for detecting Au- and Pt-based anticancer drugs.^2,23,24^ However, those studies have hitherto been restricted to adherent cell lines under cell culture conditions, and the potential of NanoSIMS in pharmacology is hence far from being fully exploited.

Here we applied a combinatorial approach using NanoSIMS and transmission electron microscopy (TEM)^25–28^ that allows the parallel investigation of cell ultrastructure and drug distribution in samples obtained from mice treated with therapeutically relevant doses of experimental platinum(iv) drugs. Moreover, laser ablation inductively coupled plasma mass spectrometry (LA-ICP-MS) was applied for region preselection. Together, this workflow enables multi-scale imaging that allows estimation of relative platinum concentrations and spatial distribution within the same tissue material on both the supra- and subcellular scale.

## Experimental

### Chemicals

Milli-Q water (18.2 MΩ cm, Milli-Q Advantage, Darmstadt, Germany) was used for all dilutions for ICP-MS measurements. Nitric acid (≥65%, p.a., Fluka, Buchs, Switzerland) was further purified in a quartz sub-boiling point distillation unit (Milestone-MLS GmbH, Leutkirch, Germany) before usage. Platinum and rhenium standards for ICP-MS measurements were purchased from CPI International (Amsterdam, The Netherlands). Tissue-Tek medium (Sakura Finetek, The Netherlands) was used for embedding of the cryosections. All other reagents and solvents were obtained from commercial sources and were used without further purification. Complexes 1 and 2 were synthesized according to literature.^8,13^

### General procedures

Animal experiments were approved by the local ethics commission and carried out according to the Austrian and FELASA guidelines for animal care and protection. Six- to eight-week-old Balb/c mice were purchased from Harlan Laboratories (San Pietro al Natisone, Italy). The animals were kept in a pathogen-free environment, and every procedure was done in a laminar airflow cabinet. Murine CT-26 cells (5 × 10^5^) were injected subcutaneously into the right flank, and therapy was started when tumor nodules were palpable (day 4). Mice were treated with compound 1 (i.p. 8.5 mg kg^−1^ in H_2_O at days 4, 7, 11 and 13) or compound 2 (i.v. 18 mg kg^−1^ in 0.9% NaCl solution once at day 4). A cisplatin-treated mouse (referred to in Table 1) was subjected to the maximum tolerated dose of the drug (3 mg kg^−1^ in 5% glucose solution at days 4, 7, 11 and 14). Animals were controlled for distress development every day, and tumor size was assessed regularly by caliper measurement. Mice were anesthetized 24 h after the last drug application (day 14 in case of 1, and day 5 in case of 2), and organs were dissected. For LA-ICP-MS measurements and quantitative platinum determination by solution-based ICP-MS, organ parts of the respective mouse were shock-frozen in liquid nitrogen and kept at −80 °C until analysis. The corresponding organ parts were immediately chemically fixed for NanoSIMS investigations.

#### ICP-MS measurements

Quantification of platinum in liquid samples was carried out with an ICP-quadrupole MS instrument Agilent 7500ce (Agilent Technologies, Waldbronn, Germany). The ICP-MS instrument was equipped with a CETAC ASX-520 autosampler (Nebraska, USA) and a MicroMist nebulizer at a sample pump rate of approx. 0.25 ml min^−1^. The instrument was tuned on a daily basis. Rhenium served as internal standard for platinum to account for instrumental fluctuations and matrix effects. The ICP-MS was equipped with nickel cones and operated at an RF power of 1550 W. Argon was used as plasma gas (15 L min^−1^) and as carrier gas with a flow rate of ∼1.1 L min^−1^. The dwell time was set to 0.3 s, and the measurement was performed in 10 replicates. The Agilent MassHunter software package (Workstation Software, Version B.01.01, 2012) was used for data processing. Digestion of tissue samples (approx. 10–30 mg) was performed with sub-boiled nitric acid by using a microwave system Discover SP-D (CEM Microwave Technology, Germany). The following microwave parameters were used: temperature: 200 °C; ramp time: 4 min; hold time: 6 min; maximal power: 300 W. Digested samples were diluted with Milli-Q water, resulting in nitric acid concentrations lower than 3% and platinum concentrations lower than 20 ng g^−1^.

#### Quantitative bioimaging in tissue samples by LA-ICP-MS

For LA-ICP-MS measurements, tumor and kidney samples were embedded in Tissue-Tek medium and cryosectioned into slices of 20 μm thickness with a cryotom (Microm HM 550, Thermo Fisher). Quantitative bioimaging by LA-ICP-MS was performed according to a previously described procedure, using matrix-matched calibration standards.^29^ A Nd:YAG solid state laser (NWR 213, ESI, Fremont, CA, USA) at a wavelength of 213 nm was used to obtain the spatially-resolved distribution of platinum in tumor and kidney sections. Laser ablation was performed as described previously.^9^ Data were recorded by using a Triple Quadrupole ICP-MS Agilent 8800 instrument (Agilent Technologies, Tokyo, Japan) and processed with the Agilent MassHunter software package (Workstation Software, Version B.01.03, 2013). The software Igor Pro (Wavemetrics, Igor Pro 6.34A) together with its add-on Iolite (Iolite Version 2.5) was used for further data processing and generation of platinum distribution maps.^30^

#### Sample preparation for NanoSIMS and TEM imaging

Organs from a freshly sacrificed mouse were chemically fixed with 2.5% glutaraldehyde solution overnight at +4 °C and washed with 0.1 M sodium cacodylate buffer, pH 7.3. The tumor sample was postfixed with 1% OsO_4_ solution in 0.1 M sodium cacodylate buffer for 2 h and washed in buffer prior to dehydration. Dehydration was performed in an ascending ethanol series (30%, 50%, 70%, 90% and 2× 100% ethanol, 5–10 min each). Ethanol was then replaced with pure acetone, which was substituted gradually with low viscosity resin (Agar Scientific, UK). Samples infiltrated with pure resin were placed in embedding molds and polymerized overnight at 60 °C.

Resin sections of embedded kidney were cut by using an ultramicrotome (LEICA Ultracut S, Germany). For cutting semi-thin sections, 300 nm in thickness, freshly prepared glass knives were used. 5 to 10 sections were transferred with an eyelash onto a droplet of bidistilled water located on indium tin oxide (ITO) coated glass slides (7 × 7 × 1 mm^3^, Präzisions Glas & Optik GmbH, Iserlohn, Germany). A wrinkle-free attachment of the sections to the glass slide was achieved by drying on a warm plate.

The resin block with the tumor sample was cut with a diamond knife (Diatome, Switzerland) to obtain 4 consecutive ultra-thin sections of 100 nm thickness. Sections 1 and 4 were placed on 200 mesh copper grids counterstained with uranyl acetate and lead citrate and imaged in a TEM ZEISS Libra 120 (Germany) at 120 kV. Images were acquired by using a bottom stage digital camera and iTEM software (Soft Imaging System GmbH, Münster, Germany). Sections 2 and 3 were placed on an ITO coated glass slide. One of the adherent sections was subsequently analyzed with NanoSIMS.

#### NanoSIMS analysis

NanoSIMS measurements were carried out on an NS 50L instrument (Cameca, France). The detectors of the multicollection assembly were positioned to enable parallel detection of ^12^C_2_^−^, ^12^C^14^N^−^, ^31^P^−^, ^34^S^−^, ^190^Os^−^ and ^195^Pt^−^ secondary ions. The inner width of the selected spectrometer entrance slit was 20 μm (“ES#3”), and a 150 μm aperture slit (“AS#2”) was inserted in the secondary ion beam path to reduce beam divergence. The electrostatic lenses and deflectors inside the spectrometer were adjusted to achieve a mass resolving power (MRP) of >7500 (according to Cameca's definition) for detection of ^195^Pt^−^ secondary ions. Spectrometer tuning, mass calibration and detector (electron multiplier) calibration were carried out on a semi-thin section of resin-embedded SW480 cells after precipitation of a finely grained cisplatin deposit obtained from spotting and evaporation of an aqueous, 1 mM cisplatin solution on the sample surface.

All data were acquired as multilayer image stacks obtained by sequential scanning of a finely focused Cs^+^ primary ion beam over areas between 35 × 35 and 70 × 70 μm^2^ with 512 × 512 pixel image resolution. The physical resolution (probe size) was approx. 80 nm, accomplished through beam divergence reduction by insertion of a diaphragm with 200 μm inner diameter (“D1#3”) in the primary ion beam path. The per pixel dwell time of the primary ion beam was 10 ms, and the number of image (scanning) cycles was chosen to achieve a total dwell time of 140 to 200 ms per pixel. Between every image cycle, secondary ion beam drift was corrected by automatic beam centering and coaxial lens (“EOS”) voltage optimization (utilizing the ^12^C_2_^−^ signal as reference) as well as automatic peak centering for each of the recorded secondary ion species. The total acquisition time was in the range from 15 to 20 h per measurement.

#### Image processing, numerical data evaluation and statistics

NanoSIMS image data were evaluated by using the WinImage software package (version 2.0.8) provided by Cameca. Prior to stack accumulation, the individual images were aligned to compensate for positional variations arising from primary ion beam and/or sample stage drift. Secondary ion signal intensities were dead-time corrected on a per-pixel basis. Individual ROI, representative for distinct cellular compartments, were defined manually, based on the morphological features identifiable in the relative N, P, and S elemental distribution, as inferred from the ^12^C^14^N^−^, ^31^P^−^, and/or ^34^S^−^ signal intensity distribution maps. ROI-specific ^12^C_2_^−^-normalized ^195^Pt^−^ intensity values were analyzed for normal distribution (Kolmogorov–Smirnov test, *p* < 0.05). Normally distributed data were tested for significant differences in the arithmetic means by application of Welch's *t*-test, and not normally distributed datasets were tested for significant differences in the medians by application of the Mann–Whitney *U*-test. All statistical calculations were conducted with the GraphPad Prism 5.0 software (GraphPad Software Inc, USA). For the correlation of images derived from TEM and NanoSIMS, GIMP 2.8 software (GNU Image Manipulation Program, freeware) was employed.

## Results and discussion

### Platinum distribution in kidney

**Fig. 2:**
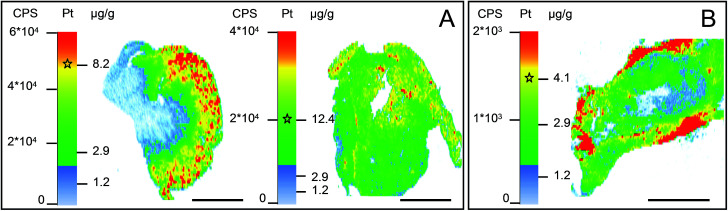
LA-ICP-MS-determined local concentrations of platinum in (A) a kidney (left) and liver (right) tissue section of a mouse upon treatment with compound 1; (B) malignant tissue (CT-26 tumor) section of a mouse upon treatment with compound 2. The asterisks within the intensity scales indicate the average platinum concentration determined by ICP-MS after microwave-assisted digestion. Scale bars = 2 mm.

An individual tumor-bearing mouse with subcutaneously injected CT-26 colon cancer cells was subjected to intraperitoneal treatment with compound 1 for two weeks. The animal was sacrificed 24 h after the last injection and organs were collected immediately. Tumor, kidney and liver were dissected in halves and the resulting parts were separately (i) shock-frozen for the ICP-MS platinum accumulation measurements and LA-ICP-MS imaging studies and (ii) fixed for NanoSIMS investigations. The total platinum content in tumor, kidney, and liver tissues was assessed with ICP-MS (Table 1). Subsequently, LA-ICP-MS was conducted on the organs with the highest platinum accumulation to obtain quantitative information on the spatial platinum distribution. LA-ICP-MS analyses showed a rather homogeneous platinum distribution in liver tissue and predominant platinum accumulation in the cortical structures of the kidney (Fig. 2A). Compound 1 yielded 8- to 10-fold higher platinum concentrations in the cortex than in the medulla, corresponding to ∼7–10 μg g^−1^ platinum, which was one of the highest local concentrations of platinum in the investigated samples. The finding of predominant cortical localization of platinum from compound 1 is in good accordance with previous LA-ICP-MS studies in kidney sections upon treatment with cisplatin, oxaliplatin, and satraplatin.^9,29,31,32^

**Fig. 3:**
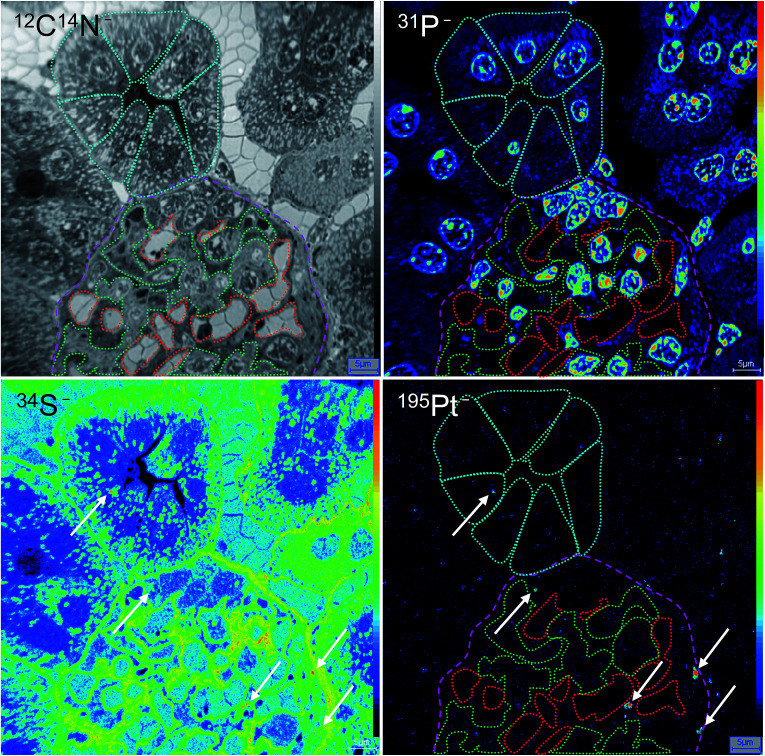
Platinum distribution in mouse kidney upon treatment with compound 1. NanoSIMS ^12^C^14^N^−^, ^31^P^−^, ^34^S^−^, and ^195^Pt^−^ secondary ion signal intensity distribution images show the subcellular platinum localization in a semi-thin section of a murine kidney cortex. Colored regions of interest (ROI) refer to glomerulus (magenta), proximal convoluted tubule cells (cyan), mesangial cells (green), and capillaries (red). Arrows show platinum hotspots co-localized with sulfur-rich cytoplasmic organelles in podocytes and cells of the tubule. Intensities are displayed on a false color scale, ranging from low intensities (black) to high intensities (red/white). Scale bars = 5 μm.

**Fig. 4:**
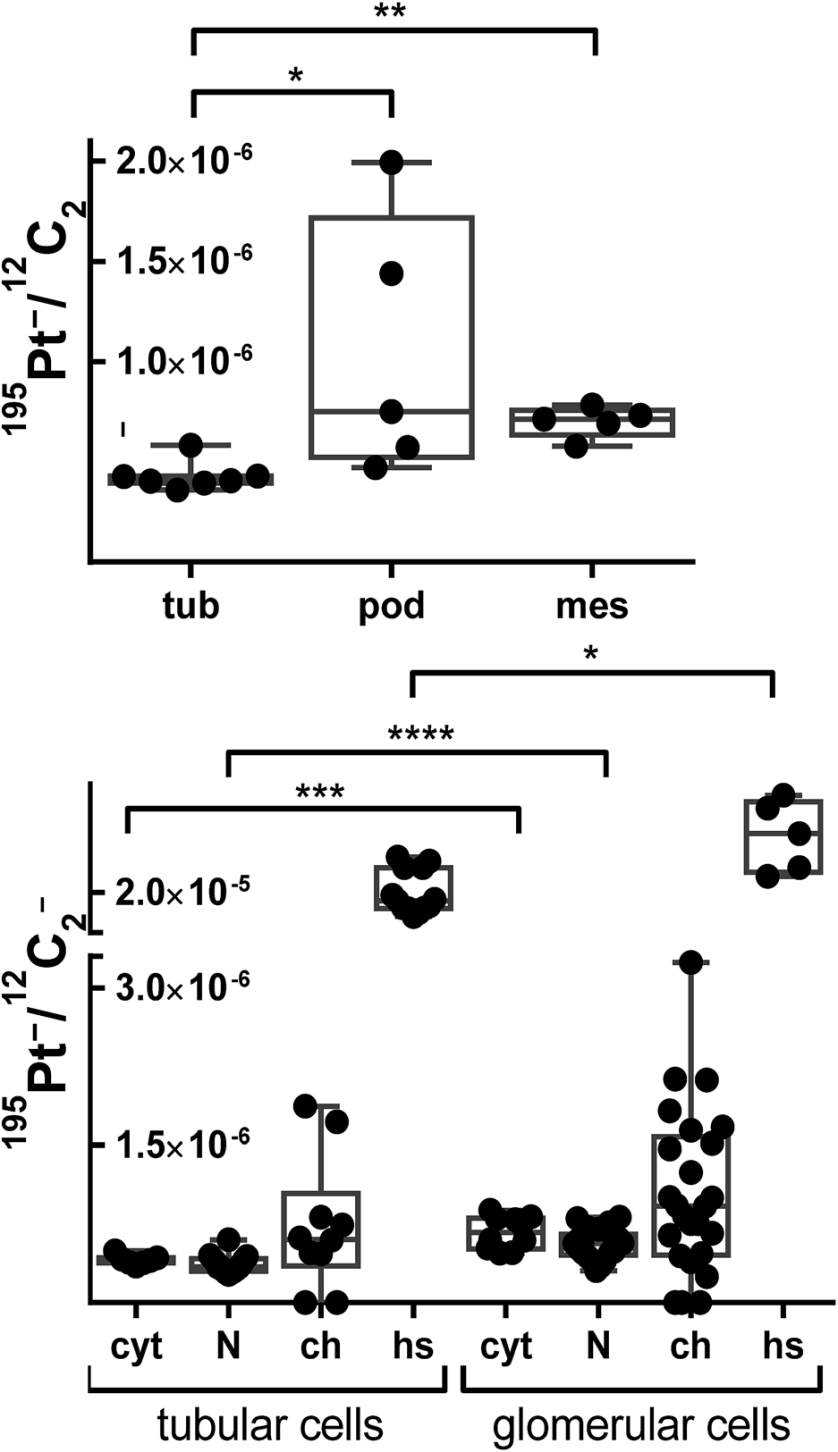
Relative platinum content in different mouse kidney cell types (top) and average relative platinum accumulation in different cell compartments (bottom) upon treatment with compound 1. Data were inferred from ROI specific evaluation of ^12^C_2_^−^ normalized ^195^Pt^−^ secondary ion signal intensity images. Data points refer to individual ROI values. The box-and-whisker plots display the extreme values (min/max), median and lower/upper quartiles. Asterisks refer to the significance level with *p < 0.05, **p < 0.01, ***p < 0.001, ****p < 0.0001. Labels: tub – proximal tubule cells; pod – podocytes; mes – mesangial cells; cyt – cytoplasm; N – nucleus; ch – chromatin; hs – platinum hotspots/aggregations.

In general, the platinum distribution in renal tissue is of high relevance as nephrotoxicity is one of the dose-limiting side effects of cisplatin therapy observed in the clinical routine.^33,34^ Overall, the relation between kidney functionality and platinum-based chemotherapy is very complex,^35,36^ especially as the kidney has plenty of functions, including besides its excretory function also the preservation of fluid, electrolyte and acid–base balance as well as the regulation of blood pressure.^37^ Consequently, the kidney is characterized by a very specific organ structure, containing a variety of highly specialized cell types. To better understand cellular triggers responsible for nephrotoxicity of platinum drugs *in vivo*, novel tools are required to gain deeper insights into these processes that in turn may help to improve clinical interventions. ^12^C^14^N^−^, ^31^P^−^ and ^34^S^−^ secondary ion signal intensity distribution images, indicating the distribution of cellular proteins, nucleic acids and basement membrane, respectively, were used to distinguish between different structures of the renal cortex. Simultaneously acquired ^195^Pt^−^ images were then used to study the platinum distribution. As shown in Fig. 3, the screened area comprised a part of glomerulus surrounded by renal tubules. The high levels of sulfur of the basement membrane (revealed in secondary ion maps) were used to define the cell types of the glomerulus (Fig. 3, labeled in magenta): The endothelial cells of the capillaries (indicated in red) together with mesangial cells (green) are separated by the basement membrane from the podocytes that are forming the renal network on the urinary side of the glomerular filtration barrier. In contrast to the endothelium, mesangial cells are characterized by a pronounced cytoplasmic content, which is highlighted in ^12^C^14^N^−^ as well as ^34^S^−^ signal intensities. In addition, the structure lying above the glomerulus was classified as the proximal convoluted tubule (cyan) according to the characteristic tall cuboidal epithelium morphology. Interestingly, a scattered ^195^Pt^−^ intensity distribution was observed in all cortical structures. The average platinum levels in cells of the glomerulus (mesangial cells and podocytes) were shown to be significantly higher than in the tubules (*p* < 0.05, Mann–Whitney *U*-test, Fig. 4, top), although the distribution pattern between different compartments of tubular and glomerular cells varied to similar extents (Fig. 4, bottom). Platinum in the glomerulus was found to be prone to aggregation in sulfur-rich organelles of the podocytes rather than of mesangial cells. In addition, cells of the proximal convoluted tubule were also shown to accumulate platinum in sulfur-rich organelles. Notably, both, proximal tubular cells as well as podocytes, were reported to have a prominent lysosomal compartment including endosomes and lysosomes^37^ that might be responsible for cytoplasmic accumulation of platinum. These findings are in good agreement with a generally high affinity of platinum to sulfur^38^ and the known effect of metallodrugs on the lysosomal compartment of animal tissues.^39^ Both proximal tubular cell damage^40,41^ as well as inhibition of glomerular filtration^42,43^ have been previously suggested as possible nephrotoxic consequences of platinum drug treatment. Our findings, albeit inferred from one individual mouse only, reveal that various cortical cell types might be affected upon platinum(iv) drug treatment but result in different cell responses (*e.g.* cytoplasmic aggregation in podocytes and more even distribution in mesangium). Thus, NanoSIMS demonstrated high sensitivity and spatial resolution, aiding in renal structure recognition and identification of different cell types accumulating platinum species.

### Platinum distribution in tumor tissue

For visualizing and quantifying platinum distribution at the cellular and subcellular level in murine samples, unambiguous identification of cell borders and small organelles is necessary. NanoSIMS secondary ion signal intensity distribution images related to the (abundant) elements N, P, and S can be readily used to distinguish the more prominent subcellular structures (*e.g.*, nucleus, nucleolus, chromatin). However, the exact identification of small organelles remains highly challenging (when judged only by morphological appearance in distribution maps of elements) and, thus, requires the application of complementary techniques such as transmission electron microscopy (TEM). Electron microscopy techniques were combined with SIMS before for applications in microbial and cell biology.^25–28,44^ However, the majority of these studies were applied to conventional cell culture samples.

For these investigations, an individual CT-26 tumor-bearing mouse was subjected to intravenous treatment with compound 2 for 24 h. Intravenous application of this drug is necessary to allow its binding to serum albumin, which is a prerequisite for its tumor-targeting behavior. The average platinum levels upon single administration of compound 2 in the tumor (determined by ICP-MS) were ∼4 μg g^−1^ (Table 1). Pre-characterization by LA-ICP-MS bioimaging indicated that the tumor sample exhibited local platinum concentrations ranging from around 1 to 6 μg g^−1^ (Fig. 2B). Platinum enrichment (5–6 μg g^−1^) could be mainly observed in the outer regions of the tumor section. Due to the strong affinity of the drug to serum proteins, it is very likely that this corresponds to the localization of blood (micro)vessels. In accordance, the necrotic center of the tumor accumulated platinum to a lower extent (1.5–3 μg g^−1^). Overall, the platinum distribution is consistent with the concept of accumulation in the malignant tissue based on the enhanced permeability of the vascular system and retention of macromolecules in solid tumor tissue (EPR effect).^14^ Notably, despite the clinical investigation of a few albumin-bound drugs (aldoxorubicin, albumin-bound paclitaxel),^15,16^ this is the first study showing the intratumoral distribution of a compound targeting tumors by albumin-mediated delivery.

**Fig. 5:**
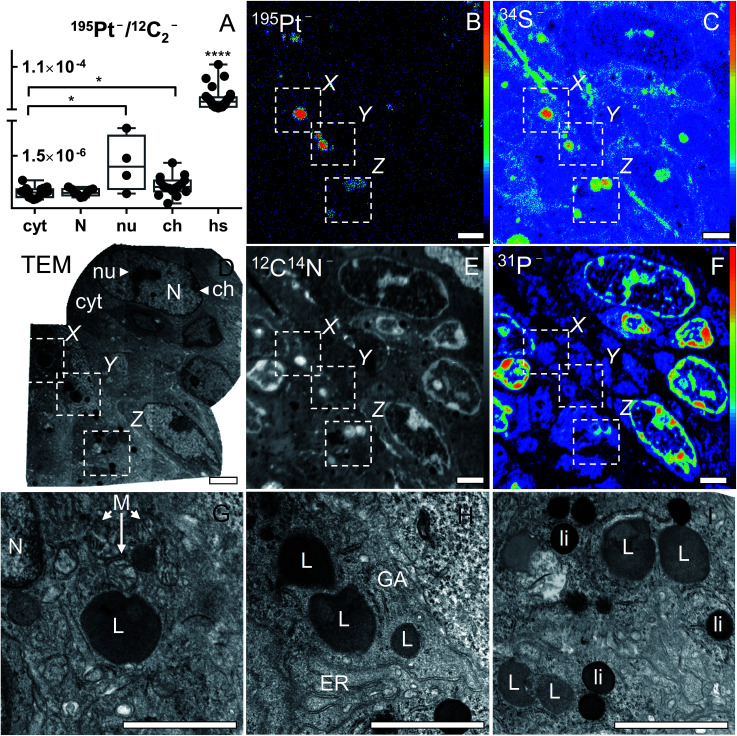
Platinum distribution in cancer cells from a CT-26 tumor-bearing mouse upon treatment with compound 2. Asterisks refer to the significance level with *p < 0.05 and ****p < 0.0001. NanoSIMS ^195^Pt^−^, ^34^S^−^, ^12^C^14^N^−^ and ^31^P^−^ secondary ion maps (B, C, E and F) show the subcellular localization of the elements in an ultra-thin section of malignant tissue. Intensities are displayed on a false color scale ranging from low intensities (black) to high intensities (red/white). TEM micrographs show the ultrastructure of the corresponding area from a consecutive resin section: overview (D); magnification of platinum-accumulating structures (hotspots) confirming the platinum co-localization with S-rich organelles of lysosomal origin (G, H and I). Field of views in TEM images shown in panels G, H and I correspond to *X*, *Y*, and *Z* areas in panels B–F, respectively. Labels: N – nucleus; nu – nucleolus; ch – chromatin; cyt – cytoplasm; hs – platinum hotspots; M – mitochondria; L – lysosomes; ER – endoplasmic reticulum; GA – Golgi apparatus; li – osmiophilic lipid droplets. Scale bars = 2 μm.

For the corresponding NanoSIMS analysis, a separate part of the same tumor was chemically fixed with 2.5% glutaraldehyde solution and additionally stained with OsO_4_ for TEM prior to resin embedding. TEM was employed on a 100 nm section to identify regions of interest that were subsequently analysed by NanoSIMS imaging. The ultrastructure of cells was thoroughly investigated prior to elemental imaging, and the TEM images were employed for correlative co-localization analyses. The NanoSIMS measurement was conducted on a consecutive 100 nm thin slice. Based on ^12^C^14^N^−^, ^31^P^−^ and ^34^S^−^ secondary ion maps, cells of the malignant tissue were identified (Fig. 5B, C and F). For quantitative analysis, the cellular compartments were manually defined based on characteristic features discernible in the secondary ion images as follows: (I) cytoplasm (without hotspots/platinum aggregations), including the cell area outside the nucleus; (II) nucleus, including phosphorus-rich chromatin structures and nuclear matrix, but excluding the nucleolus; (III) nucleolus, round shaped N-, P- and S-rich structure inside the nucleus; (IV) chromatin, dense phosphorus-rich (but not sulfur-rich) unevenly distributed regions along the inner side of the nuclear membrane; and (V) cytoplasmic platinum aggregations highlighted in platinum signal intensity (Fig. 5). ROI-specific analyses of the ^12^C_2_^−^-normalized ^195^Pt^−^ signal intensity distribution revealed similar accumulation of platinum in nucleic and cytoplasmic compartments (Fig. 5A). However, local platinum levels in nucleoli and chromatin structures turned out to be higher than the average cytoplasmic values (*p* < 0.05; Mann–Whitney *U*-test and Welch's *t*-test, respectively). In the cytoplasm, platinum was aggregated in relatively small sulfur-rich organelles, most of which were also rich in proteins (according to CN^−^ signal intensity distribution). Superposition of NanoSIMS and TEM images obtained from consecutive slices was used to identify the sulfur-rich organelles responsible for platinum accumulation. Based on the size (up to 1 μm in diameter), morphology (spherical cytoplasmic structures with visible membrane) and their characteristic dark grey color differing clearly from the blackening of strongly osmiophilic lipid droplets, we classified those organelles as lysosomes (Fig. 5G–I).

Very little ultrastructural information is available on the intracellular distribution of platinum drugs. In previous electron and immunoelectron microscopy studies, cisplatin was found to accumulate to a different extent in both cytoplasmic and nuclear structures of cancer cells.^45,46^ However, electron microscopy-based techniques are limited to the imaging of pronounced platinum accumulations or DNA–platinum adducts (in case of immunolabeling), therefore missing the information on other cellular targets and non-aggregated drug. In good accordance with our data, loci with high-density chromatin and nucleoli were reported among the nuclear targets of cisplatin. In the cytoplasm, freely scattered distribution of the drug^45^ as well as accumulation in mitochondria was shown.^46^ The absence of lysosomal localization in these studies can be explained by a short exposure time^45^ and by limitation of immuno-based electron microscopy to DNA–platinum adducts detection.^46^

Two hypotheses can be formulated to explain the lysosomal localization of platinum hotspots from compound 2. Firstly, compound 2 might be released and reduced to oxaliplatin extracellularly and subsequently bind to sulfur-rich parts of the cell such as the lysosomes due to the affinity of platinum(ii) drugs to sulfur. Alternatively, compound 2 might be taken up in its albumin bound form by lysosomes, followed by albumin degradation, reduction to oxaliplatin and binding to lysosomal sulfur donors. With regard to the first hypothesis, the Lewis acid “hardness/softness” of metal ions is known to distinctly impact on their intracellular behavior and, consequently, biological activity.^47^ Thus, in case of the very “soft” platinum(ii) ions, a distinct affinity for sulfur is well known.^38,48^ In accordance, sulfur-containing molecules such as glutathione play an important role in the metabolism of platinum(ii) drugs. Our prior studies on cancer cell lines indicated co-localization of platinum with sulfur-rich structures inside the cells.^2^ These structures were further identified as lysosomes by the combined application of NanoSIMS and fluorescence microscopy. This finding may be particularly relevant as altered drug distribution (enhanced drug sequestration to specific cytoplasmic structures) has been recognized as one of the mechanisms underlying resistance to platinum drugs.^49–52^ Recently, the crucial role of copper transporters in platinum translocation was confirmed.^53^ Overexpression of the copper transporters ATP7A and ATP7B in the plasma membrane and trans-Golgi network (lysosomal predecessor) has been reported to confer resistance in the case of cisplatin.^54^ For example, cells transfected with ATP7B have been shown to accumulate less platinum, while cells transfected with ATP7A were found to be resistant to platinum(ii) drugs as a result of increased sequestration into cytoplasmic vesicles.^55,56^ Our results are also in good accordance with a number of fluorescence microscopy studies on platinum complexes coupled to fluorophores: with such compounds several research groups have shown platinum accumulation in acidified organelles of trans-Golgi origin: secretory export pathway vesicles,^57,58^ endocytic vesicles^59^ and lysosomes.^60,61^

With regard to the second hypothesis, there are so far no literature data available, which address the exact localization of drug release from albumin-bound prodrugs. In case of aldoxorubicin, where doxorubicin is released from albumin after acid-dependent cleavage, it can be hypothesized that drug release might be an extracellular process.^62^ However, in a mouse sarcoma model (C57/RL6J), radioactively labeled albumin was found to a high extent in the lysosomes of the tumor tissue, and there is increasing evidence that cancer cells have a very high albumin degradation rate.^63^ In general, it is still unexplored whether platinum(iv) drugs are reduced (and hence activated) intra- or extracellularly. However, we see a rather rapid accumulation of compound 2 in the malignant tissue and observe platinum in lysosomal compartments already 24 h after treatment. This might support the assumption that in case of compound 2 activation by reduction is an intracellular process.

## Conclusions

Current knowledge about subcellular accumulation and distribution of platinum drugs has mainly been derived from studies involving adherent cell cultures *in vitro*.^24,46,64^ However, this approach is based on single cells in monotypic cultures that are generally exposed to uniform conditions.^52^ In contrast, the tumor microenvironment is heterogeneous and the tissue is exposed to large concentration gradients due to extravascular drug penetration.^65^ Therefore, there is a big need for a quantitative imaging technique that can help to understand the behavior of drugs in tissues when applied to animals in therapeutically relevant concentrations. In this study, we have demonstrated that the combination of LA-ICP-MS, NanoSIMS and TEM is suitable for investigating the platinum distribution in tissue samples obtained from mice treated with two platinum(iv)-based anticancer compounds. The sensitivity of the method was proven to be sufficient for trace drug analyses in tumors and organ tissues. Specific subcellular localization of the drugs was detected both in murine tumor and kidney samples, demonstrating the utility of the approach for the elucidation of possible drug targets. Platinum-based drugs continue to be one of the most important classes of chemotherapeutic agents applied in clinical cancer therapy. Nevertheless, there is still a big need for improvement in terms of optimizing tumor selectivity and minimizing side effects. Deeper insights into subcellular trafficking and processing of platinum complexes may inspire the synthesis of novel compounds with fine-tuned properties. In the future, the reported approach might be evaluated for its clinical applicability, for example for the investigation of biopsies of patients with different responsiveness to platinum-based chemotherapy.

## Abbreviation list

CCD: Charge-coupled device
EPR: Enhanced permeability and retention
GIMP: GNU image manipulation program
FELASA: Federation of European laboratory animal science associations
HSA: Human serum albumin
ITO: Indium tin oxide
LA-ICP-MS: Laser ablation inductively coupled plasma mass spectrometry
MRP: Mass resolving power
NanoSIMS: Nano-scale secondary ion mass spectrometry
ROI: Region(s) of interest
TEM: Transmission electron microscopy

## Supplementary Material
